# Coverage Layer Phase Composition-Dependent Photoactivity of One-Dimensional TiO_2_–Bi_2_O_3_ Composites

**DOI:** 10.3390/nano10051005

**Published:** 2020-05-25

**Authors:** Yuan-Chang Liang, Kai-Jen Chiang

**Affiliations:** Department of Optoelectronics and Materials Technology, National Taiwan Ocean University, Keelung 20224, Taiwan; ssdcjh972003@yahoo.com.tw

**Keywords:** One-dimensional structure, Composites, Heterogeneous junction, Synthesis, Morphology, Photoactivity

## Abstract

TiO_2_–Bi_2_O_3_ composite rods were synthesized by combining hydrothermal growth of rutile TiO_2_ rod templates and sputtering deposition of Bi_2_O_3_ thin films. The TiO_2_–Bi_2_O_3_ composite rods with β-Bi_2_O_3_ phase and α/β-Bi_2_O_3_ dual-phase decoration layers were designed, respectively, via in situ radio-frequency magnetron sputtering growth and post-annealing procedures in ambient air. The crystal structure, surface morphology, and photo-absorption performances of the pristine TiO_2_ rods decorated with various Bi_2_O_3_ phases were investigated. The crystal structure analysis reveals that the crystalline TiO_2_–Bi_2_O_3_ rods contained β-Bi_2_O_3_ and α/β-Bi_2_O_3_ crystallites were separately formed on the TiO_2_ rod templates with different synthesis approaches. The morphology analysis demonstrates that the β-Bi_2_O_3_ coverage layer on the crystalline rutile TiO_2_ rods showed flat layer morphology; however, the surface morphology of the α/β-Bi_2_O_3_ dual-phase coverage layer on the TiO_2_ rods exhibited a sheet-like feature. The results of photocatalytic decomposition towards methyl orange dyes show that the substantially improved photoactivity of the rutile TiO_2_ rods was achieved by decorating a thin sheet-like α/β-Bi_2_O_3_ coverage layer. The effectively photoinduced charge separation efficiency in the stepped energy band configuration in the composite rods made from the TiO_2_ and α/β-Bi_2_O_3_ explained their markedly improved photoactivity. The TiO_2_-α/β-Bi_2_O_3_ composite rods are promising for use as photocatalysts and photoelectrodes.

## 1. Introduction

One-dimensional rods have been widely investigated for various binary oxides in photoactive applications because of their high surface-to-volume ratio and possibility for integration into diverse semiconductor nanodevices [1,2,3,4]. TiO_2_ shows many excellent characteristics, such as low toxicity, easy fabrication, low cost, high stability and high photosensitivity [5]. As a result, it has been widely applied in photodegradation of organics and photocatalytic water splitting [3,6]. Compared to most commonly used TiO_2_ nanoparticles or thin films, a vertically oriented TiO_2_ nanorod prepared by a simple hydrothermal method possesses high photoactivity owing to its large surface area and excellent electron transport property and is promising for integration into diverse functional nanodevices [1,3]. However, the TiO_2_ with a wide band gap was only excited by ultraviolet light contributing only 6% of the solar spectrum; moreover, the photogenerated charge carriers in the TiO_2_ easily recombine, which adversely affects the practical photoactivated performance of the TiO_2_. Recently, one-dimensional TiO_2_ oxide coupled with other narrow wide band gap materials with a suitable band alignment was revealed as a promising approach to improve the intrinsic photoactivity of the TiO_2_ [3,7]. In particular, construction of oxide heterostructures with a type II band alignment is advantageous to suppress the recombination of photogenerated electron-hole pairs and improve the photocatalytic efficiency because of formation of an inner electric field at the heterointerface [8,9]. For example, the photoactivity of the sputtering-assisted decoration of ZnFe_2_O_4_ crystallites onto TiO_2_ nanorods improves the photodegradation performance towards methylene orange (MO) [3]. Furthermore, electrospinning-derived TiO_2_-WO_3_ nanofibers show the higher activity towards photodegrading MO dyes than that of the pristine TiO_2_ in ultraviolet (UV) light [7]. These examples visibly demonstrate heterogeneous TiO_2_ based hybrids could obviously suppress the recombination of photogenerated electron-hole pairs by transferring photogenerated electrons and holes in the heterojunctions, prolong their lifetime, and significantly enhance the photoactivity.

Bi_2_O_3_ is the simplest Bi-based oxide, and is used in extensive photoactivated applications due to its excellent properties [10,11]. The Bi_2_O_3_ has a direct band gap with a wide range from approximately 2.0~3.9 eV and several polymorphs [12]. The α-Bi_2_O_3_ and β-Bi_2_O_3_ have been reported to exhibit desirable photocatalytic activity to decompose organic pollutants under irradiation [13]. Therefore, integration of Bi_2_O_3_ into TiO_2_ to form a heterogeneous structure is a potential target system to investigate possible improvement in photodegradation efficiency towards organic pollutants. Recent work by Huo et al. synthesized Bi_2_O_3_/TiO_2_ film by sol-gel method and the degradation rate of MO achieved 70% at the given irradiation condition [14]. Other methods such as chemical bath deposition and dip-coating were also reported to have prepared TiO_2_–Bi_2_O_3_ composites for the purpose of photocatalytic and other applications [15,16]. However, these chemical solution routes are disadvantageous to modulate the phase content of Bi_2_O_3_ and this hinders the design of the suitable Bi_2_O_3_ polymorphs to be integrated into the one-dimensional TiO_2_ system with desirable photocatalytic functionality. Herein, we initially present a sputtering-assisted deposition technique to deposit Bi_2_O_3_ crystals with β-Bi_2_O_3_ phase and α/β-Bi_2_O_3_ dual phase as decoration thin layers. The Bi_2_O_3_ coverage layer phase composition, microstructures, and resultant effects in photoactivated performance of the TiO_2_–Bi_2_O_3_ composite rods are correlated in this study. The results herein show control of the Bi_2_O_3_ coverage layer phase composition via sputtering-assisted deposition is a promising method to design and tune one-dimensional TiO_2_–Bi_2_O_3_ composites with a desirable photoactivity.

## 2. Materials and Methods

One-dimensional aligned TiO_2_ rods were grown on F-doped SnO_2_ (FTO) glass substrates using a hydrothermal method. The detailed preparation of reaction solution for hydrothermal growth of the TiO_2_ rods has been described elsewhere [17]. Furthermore, TiO_2_–Bi_2_O_3_ composite rods were fabricated by radio-frequency magnetron sputtering Bi_2_O_3_ thin films onto the surfaces of the TiO_2_ rod templates. The bismuth metallic disc with a diameter of two inches was used as the target during the sputtering processes. The Bi_2_O_3_ coverage layers onto the surfaces of the TiO_2_ rods were fabricated through two different approaches. The first set of the Bi_2_O_3_ coverage thin layer was grown in mixed Ar/O_2_ ambient with a ratio of 1:1 at 425 °C. The working pressure during thin-film growth was maintained at 2.66 Pa and sputtering power of the bismuth metallic target was maintained at 30 W. The distance between the substrate and target is 7 cm. The second set of the Bi_2_O_3_ coverage thin layer was formed through post-annealing the sputtering deposited metallic Bi thin films at 325 °C in ambient air for 1 h. The Bi metallic thin films were grown at room temperature with a pure Ar atmosphere and transformed into Bi_2_O_3_ thin films after the post-annealing procedure. The as-synthesized TiO_2_–Bi_2_O_3_ composite rods with two different Bi_2_O_3_ coverage thin layers from in situ heating sputtering and post-annealing procedures were respectively denoted as TiO_2_/s–Bi_2_O_3_ composite rods and TiO_2_/a–Bi_2_O_3_ composite rods, in this study. The in situ sputtering formed s-Bi_2_O_3_ film has a layered coverage feature and the post-annealing formed a-Bi_2_O_3_ film has a sheet-like coverage feature on TiO_2_ templates.

Crystallographics of the samples were investigated by X-ray diffraction (XRD; D2 PHASER, Bruker, Karlsruhe, Germany) and the measurement at two theta range was set to 20°–60°. The surface features of the samples were characterized by scanning electron microscopy (SEM; S-4800, Hitachi, Tokyo, Japan), respectively. High-resolution transmission electron microscopy (HRTEM; JEM-2100F, JEOL Tokyo, Japan) was used to investigate the detailed microstructures of the TiO_2_–Bi_2_O_3_ composite rods. The attached energy-dispersive X-ray spectroscopy (EDS) was used to investigate the elemental composition of the composite rods. X-ray photoelectron spectroscopy (XPS; ULVAC-PHI XPS, ULVAC, Chigasaki, Japan) was used to investigate elemental composition of the samples. An ultraviolet–visible (UV–Vis) spectrophotometer (V750, Jasco, Tokyo, Japan) was used to investigate the reflectance spectra of various rod samples. Photodegradation experiments were performed by comparing the degradation of aqueous solution of methyl orange (MO; 10^−6^ M, 10 mL) containing various rod samples under light irradiation excited from the 100 W Xe arc lamp. For the visible light driven photodegradation tests, a UV light-cutting filter was used during photodegradation processes. The mixed suspensions were first magnetically stirred in the dark for 32 min to reach the adsorption–desorption equilibrium. Moreover, photoelectrochemical properties (PEC) were performed in a three-electrode electrochemical system, where the as-synthesized rod sample on the FTO glass was used as the working electrode, a Pt wire was used as the counter electrode, and an Ag/AgCl (in saturated KCl) electrode was used as the reference electrode in an aqueous solution containing 0.5 M Na_2_SO_4_. The active area of the working electrode was 1 cm × 1 cm. The Nyquist plots of various rod samples were measured using electrochemical impedance spectroscopy (EIS, SP150, BioLogic, Seyssinet-Pariset, France).

## 3. Results and Discussion

**Figure 1:**
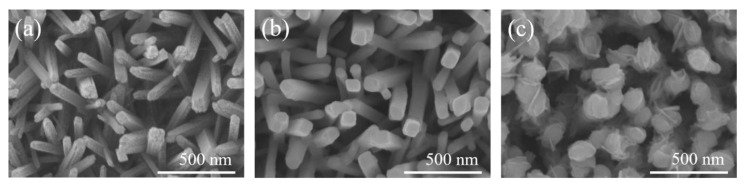
Scanning electron microscope (SEM) images: (**a**) TiO_2_ rods. (**b**) TiO_2_/s–Bi_2_O_3_ rods, (**c**) TiO_2_/a–Bi_2_O_3_ rods.

Figure 1a displays the SEM micrograph of hydrothermally derived TiO_2_ rods on the FTO substrate; these rods had a diameter in the range of approximately 80–110 nm. The TiO_2_ rods showed tetragonal prismatic morphology. The top surfaces of these rods are uneven, containing numerous up and down edge sites, whereas the sidewalls are smoother. After the sputtering deposition of the s-Bi_2_O_3_ thin film onto the TiO_2_ rods, a change in morphology was observed. The SEM image shown in Figure 1b confirms the coverage of the s-Bi_2_O_3_ thin film on the TiO_2_ rods resulted in top surfaces and sidewalls of the TiO_2_ rods becoming smooth. Figure 1c shows the SEM image of the TiO_2_ rods decorated with the a-Bi_2_O_3_ thin film. After the decoration of the a-Bi_2_O_3_ thin film onto the TiO_2_ rods, the change in surface morphology was substantial in comparison with the pristine TiO_2_ rods. The sheet-like Bi_2_O_3_ crystals were decorated onto the surfaces of the top region and sidewalls of the TiO_2_ rods, incurring undulated morphology of the TiO_2_–Bi_2_O_3_ composite rods. It was also shown that the surfaces of the ZnO-Sn_2_S_3_ nanorods exhibited undulations and a visible sheet-like crystal texture via sputtering decoration of the Sn_2_S_3_ crystals [9]. The sheet-like crystallites on the surfaces of the one-dimensional rods improved specific surface area and is beneficial in enhancing their photoactivity [9]. The SEM images evidently demonstrated that the Bi_2_O_3_ crystals were successfully coated on the surfaces of the TiO_2_ rods through a sputtering assisted method and the s-Bi_2_O_3_ and a-Bi_2_O_3_ thin films made the TiO_2_–Bi_2_O_3_ composite rods with substantially different rod surface morphologies.

**Figure 2:**
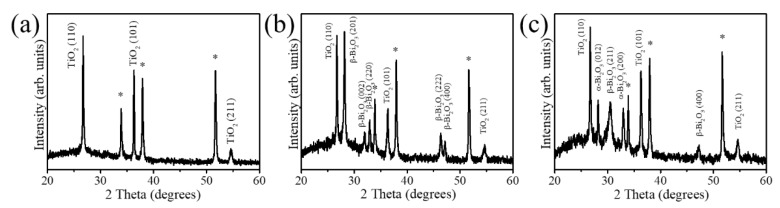
X-ray diffraction (XRD) patterns: (**a**) rutile TiO_2_ rods. (**b**) TiO_2_/s–Bi_2_O_3_ rods, (**c**) TiO_2_/a–Bi_2_O_3_ rods. The asterisks denoted Bragg reflections from the F-doped SnO_2_ (FTO) substrate.

The XRD patterns of the pristine TiO_2_ rods, TiO_2_/s–Bi_2_O_3_ composite rods, and TiO_2_/a–Bi_2_O_3_ composite rods are shown in Figure 2. In addition to Bragg reflections originated from FTO substrates in Figure 2a (marked with asterisks), distinct Bragg reflections centered at 27.4°, 36.1° and 54.3° can be indexed to (110), (101) and (211) planes of rutile TiO_2_ phase, respectively (JCPDS No.00-021-1276). The TiO_2_ rods with a good crystalline phase were formed herein. Figure 2b exhibits the XRD pattern of the TiO_2_/s–Bi_2_O_3_ composite rods. Five differentiable peaks centered at approximately 27.95°, 31.74°, 32.69°, 46.21° and 46.91° in Figure 2b can be assigned to (201), (002), (220), (222) and (400) planes of tetragonal β-Bi_2_O_3_ phase, respectively (JCPDS No.01-078-1793). The XRD result demonstrates the sputtering β-Bi_2_O_3_ thin film is in a polycrystalline phase. Moreover, the (201) Bragg reflection exhibited a substantially intense feature, revealing (201)-oriented crystals dominated the polycrystalline Bi_2_O_3_ thin film decorated onto the surfaces of the rutile TiO_2_ rods in this study. A similar (201)-orientation dominated polycrystalline β-Bi_2_O_3_ has been observed in β-Bi_2_O_3_ nanoparticles with an average grain size of 100 nm synthesized by a sol-gel method [18]. Figure 2c exhibits the XRD pattern of the TiO_2_/a–Bi_2_O_3_ composite rods. The major Bragg reflections at 2θ = 28.01° and 33.24°, corresponding to the (012) and (200) planes of the α-Bi_2_O_3_ phase were observed (JCPDS No.00-041-1449), revealing formation of a well crystallized monoclinic α-Bi_2_O_3_ phase. In addition to Bragg reflections originating from the α-Bi_2_O_3_ phase, several Bragg reflections associated with the β-Bi_2_O_3_ phase were also observed in Figure 2c. When the TiO_2_ rods were decorated with a-Bi_2_O_3_ thin film, the crystalline composite rods consisted of TiO_2_ rods and the α/β polymorphic Bi_2_O_3_ crystals were formed herein.

**Figure 3:**
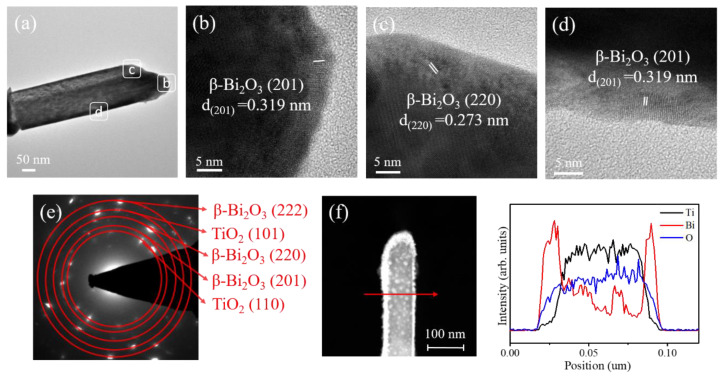
Transmission electron microscopy (TEM) analysis of the TiO_2_/s–Bi_2_O_3_ rods: (**a**) Low-magnification TEM image. (**b**–**d**) High-resolution (HR)TEM images taken from various regions of the composite rod. (**e**) Selected area electron diffraction (SAED) pattern of several TiO_2_/s–Bi_2_O_3_ rods. (**f**) Energy-dispersive X-ray spectroscopy (EDS) line scanning profiles across the composite rod.

Figure 3a shows a low-magnification transmission electron microscope (TEM) image of a single TiO_2_/s–Bi_2_O_3_ composite rod. A thin and flat β-Bi_2_O_3_ layer was homogeneously covered on the surface of the TiO_2_ rod. The high-resolution (HR) TEM images taken from the various regions of the composite rod are shown in Figs. 3b–d. The lattice fringe spacing of approximately 0.32 nm and 0.27 nm for the outer region of the composite rod corresponded to the interplanar distance of tetragonal β-Bi_2_O_3_ (201) and (220) crystallographic planes, respectively, revealing well the crystalline β-Bi_2_O_3_ phase formed on the outer region of the composite rod. However, the arrangement of lattice fringes in the inner region of the composite rod is not visibly distinguished because of the overlapped stack of the TiO_2_ and Bi_2_O_3_ oxides. Figure 3e presents the selected area electron diffraction (SAED) pattern obtained from several TiO_2_/s-Bi_2_O_3_ composite rods. It exhibited distinct diffraction spots arranged in circles with various radii. These centric diffraction patterns indicated the co-existence of the crystalline TiO_2_ and β-Bi_2_O_3_ phases, demonstrating the successful growth of the crystalline TiO_2_–Bi_2_O_3_ composite rods via sputtering decoration of β-Bi_2_O_3_ crystallites on to the surfaces of the TiO_2_ rods herein. Figure 3f displays EDS line-scanning profiles across the composite rod. The Ti element was located inside the composite rod, demonstrating the position of the TiO_2_ rod. The O element was distributed over the cross-sectional region of the whole rod. The Bi element distributed around the TiO_2_ rod, revealing the successful coverage of the Bi_2_O_3_ film on the TiO_2_. Furthermore, the corresponding HAADF-STEM image in Figure 3f shows the β-Bi_2_O_3_ crystals covered on the top region of the TiO_2_ rod were thicker than that on the lateral region of the composite rod. Moreover, the β-Bi_2_O_3_ coverage film on the lateral region of the composite rod had a thickness in the range of approximately 15–28 nm.

**Figure 4:**
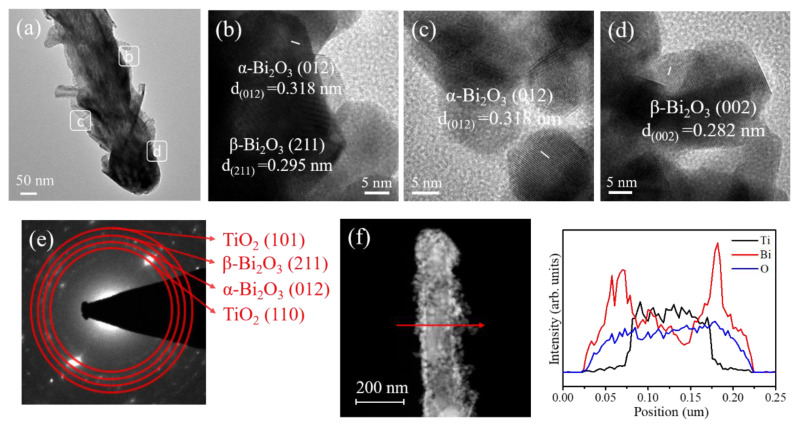
TEM analysis of the TiO_2_/a–Bi_2_O_3_ rods: (**a**) Low-magnification TEM image. (**b**–**d**) HRTEM images taken from various regions of the composite rod. (**e**) SAED pattern of several TiO_2_/a–Bi_2_O_3_ rods. (**f**) EDS line scanning profiles across the composite rod.

Figure 4a shows a low-magnification TEM image of the TiO_2_/a–Bi_2_O_3_ rod. Unlike the composite rod as displayed in Figure 3a, the Bi_2_O_3_ coverage layer exhibited a morphology of randomly oriented sheet-like aggregates consisted of numerous tiny grains. In comparison with the surface morphology of the TiO_2_/s–Bi_2_O_3_ composite rod, the surface crystal size distribution was more non-homogeneous for the TiO_2_/a–Bi_2_O_3_ composite rod. The surface of the TiO_2_/a–Bi_2_O_3_ composite rod was substantially undulated. Figure 4b–d show HRTEM images taken from the outer regions of the composite rod. Notably, many tiny grains were observed in the HR images. These tiny grains aggregated with each other to form the sheet-like crystals as exhibited in Figure 4a. Clear lattice fringes were observed in the constituent tiny grains; the lattice fringe spacing of approximately 0.318 nm is associated with lattice plane distance of the monoclinic α-Bi_2_O_3_ (012). Moreover, the lattice fringe spacing of 0.295 nm and 0.282 nm is ascribed to the crystallographic interplanar distance of the (211) and (002) of the β-Bi_2_O_3_ phase, respectively. A clear crystalline feature of the Bi_2_O_3_ crystals was exhibited in the HRTEM images. Figure 4e shows the SAED pattern of several TiO_2_/a–Bi_2_O_3_ composite rods. The visible spots arranged in centric patterns demonstrate the good crystalline quality of the composite rods. The concentric rings could be attributed to diffraction from the (110) and (101) planes corresponding to the rutile phase of TiO_2_ and the plane corresponding to the α and β phase Bi_2_O_3_. The SAED analysis herein agrees with the XRD pattern, revealing that crystalline TiO_2_-based composite rods consisted of α/β dual-phase Bi_2_O_3_ were formed herein. The cross-sectional EDS line-scanning profiles (Figure 4f) reveal the Bi signals were substantially intense in the outer region and the marked Ti signal was confined to the inner region of the composite rod, indicating that the composite rod consisted of a TiO_2_ core and a Bi_2_O_3_ coverage layer.

**Figure 5:**
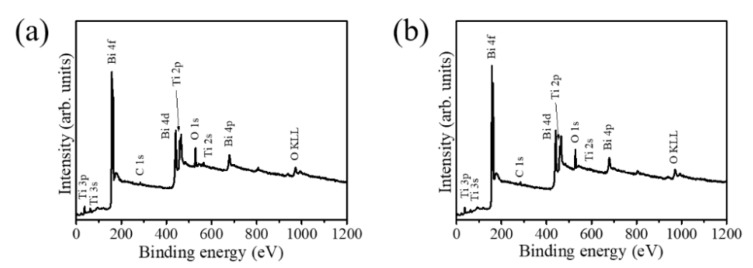
X-ray photoelectron spectroscopy (XPS) analysis: (**a**) TiO_2_/s–Bi_2_O_3_ and (**b**) TiO_2_/a–Bi_2_O_3_ rods.

Figure 5a,b displays the XPS spectra of the TiO_2_/s–Bi_2_O_3_ and TiO_2_/a–Bi_2_O_3_ composite rods, respectively. The primary peak features in the XPS spectra include the Ti, Bi, and O signals that originated from the TiO_2_–Bi_2_O_3_ composites. Notably, the carbon signal was observed herein because of the carbon surface contamination of the rod samples exposed to ambient air. Moreover, no signals from other elements were detected in the XPS spectra. The experimental results show a composite structure consisted of Ti, Bi, and O elements was formed in this study.

**Figure 6:**
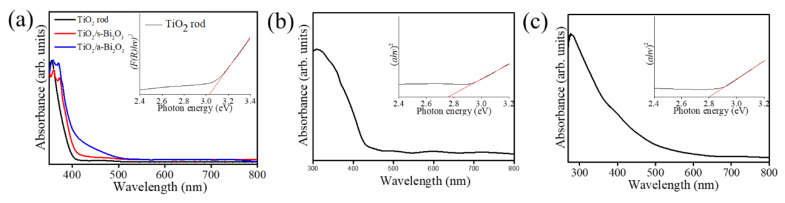
(**a**) Absorbance spectra of various rod samples. (**b**) Absorbance spectrum of the s-Bi_2_O_3_ thin film. (**c**) Absorbance spectrum of the a-Bi_2_O_3_ thin film. The insets show the bandgap of the TiO_2_ rods, s-Bi_2_O_3_ film, and a-Bi_2_O_3_ film.

The light absorption properties of the rutile TiO_2_ rods and various TiO_2_–Bi_2_O_3_ composite rods are shown in Figure 6a. The inset shows the band gap of the TiO_2_ rods is of approximately 3.03 eV by transferring Kubelka–Munk method [19]. Compared with the pristine TiO_2_ rods, the construction of the TiO_2_–Bi_2_O_3_ composite rods engendered red-shift of the absorption edge of the TiO_2_ rods. The TiO_2_–Bi_2_O_3_ composite rods exhibited a broader and stronger light absorption; the main reason for which is the synergistic absorption effect of the Bi_2_O_3_ photosensitizer and the formation of TiO_2_–Bi_2_O_3_ heterojunction [20]. The visible light band-gap energy of the Bi_2_O_3_ could lead to the broader light absorption region and induce the red shift of the absorption edge of the TiO_2_–Bi_2_O_3_ composite rods [21]. Notably, the absorption edge of the TiO_2_/a–Bi_2_O_3_ composite rods showed a more intense red shift degree than that of the TiO_2_/s–Bi_2_O_3_. The reasons might be associated with the formation of the α/β heterogeneous Bi_2_O_3_ and undulated morphology in in the decoration layer of the TiO_2_ rod surface. For the Bi_2_O_3_ films, the transmittance spectra are recorded (Figure 6b,c). The Tauc–Davis–Mott relationship is used to evaluate the bandgap of the thin film [22]. The extrapolated bandgap is approximately 2.75 and 2.80 eV for s-Bi_2_O_3_ and a-Bi_2_O_3_ thin films, respectively. Notably, the individual bandgap value of the α-phase in the a-Bi_2_O_3_ film cannot be separately evaluated in this study. The bandgap analysis herein revealed that the a-Bi_2_O_3_ film with an appearance of α-phase contributed to the blue shift of the bandgap energy from 2.75 eV to 2.80 eV in comparison with that of the pure β-phase s-Bi_2_O_3_ film (2.75 eV from Figure 6b). This result is supported with the previous reported bandgap of the α-Bi_2_O_3_ (2.85 eV) [23]. The formation of a homojunction consisted of the Bi_2_O_3_ polymorphs demonstrates a higher light harvesting ability than that of the single constituent counterpart [24]. Moreover, the sheet-like surface crystal feature in a one-dimensional composite has also been shown in several heterogeneous systems that is beneficial for light-harvesting enhancement [9,25].

**Figure 7:**
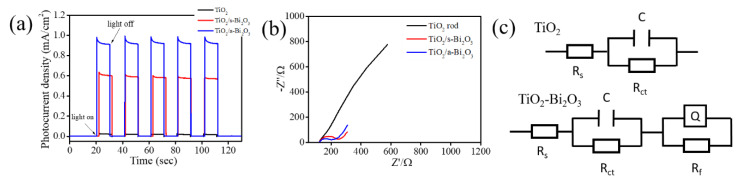
(**a**) Transient photocurrent density versus time curves of various rod samples under chopped irradiation at 1 V. (**b**) Nyquist plots of various rod samples under light irradiation. (**c**) The possible equivalent circuit used for *R*_ct_ evaluation of various rod samples.

Figure 7a displays photoresponse curves of the TiO_2_, TiO_2_/s–Bi_2_O_3_, and TiO_2_/a–Bi_2_O_3_ rods at the 1 V under chopped light irradiation. The rod samples showed rapid photoresponse and recovery properties in Figure 7a. The photocurrent density of the pristine TiO_2_ rod photoelectrode is 0.02 mA cm^−2^. Furthermore, all TiO_2_–Bi_2_O_3_ composite rods showed markedly enhanced photocurrent density with respect to the pristine TiO_2_ rods. The photocurrent density of the TiO_2_/s–Bi_2_O_3_ rod photoelectrode is approximately 0.61 mA cm^−2^ and this photocurrent density is around 30 times higher than that of the pristine TiO_2_ rod photoelectrode under irradiation. Notably, the TiO_2_/a–Bi_2_O_3_ rod photoelectrode achieved the highest photocurrent density of approximately 0.92 mA cm^−2^ in this study; this value is approximately 46 times higher than that of the pristine TiO_2_ rod photoelectrode. A substantial increase in the photocurrent density of the TiO_2_ rods sputter coated with α/β-Bi_2_O_3_ thin films is clearly demonstrated. The marked photocurrent intensity increase upon light irradiation indicates the efficient charge separation capability in the semiconductor oxides [2]. The photoresponse results herein demonstrated that the TiO_2_/a–Bi_2_O_3_ composite rods exhibited the better photoinduced electron-hole separation efficiency as compared with the TiO_2_/s–Bi_2_O_3_ rods. One of the possible reasons is associated with the suitable band alignment between the α- and β- phase Bi_2_O_3_ in the Bi_2_O_3_ coverage layer of the composite rods and type II band alignments of the TiO_2_/α- phase Bi_2_O_3_ and TiO_2_/β- phase Bi_2_O_3_ in the composite rod system. The multi-junctions in the TiO_2_/a–Bi_2_O_3_ composite rod system explained its superior electron-hole separation efficiency than the TiO_2_/s–Bi_2_O_3_ rod system in which the Bi_2_O_3_ coverage layer was in a single β phase. A substantially increased photoactivity has been shown in the multilayered ZnO/ZnS/CdS/CuInS_2_ core–shell nanowire arrays than that of the ZnO/ZnS nanowire. This is attributable to the formation of type II band aligned multi-junctions in the composite system which markedly enhances photoinduced charge separation efficiency [26]. A similar multi-junction effects has been shown in type II TiO_2_/CdS–NiO*_x_* nanorod system, in which an NiO*_x_* layer coverage on the type II TiO_2_/CdS nanorods substantially increases the photoactivity of the nanorods [27]. Moreover, in comparison with the flat s-Bi_2_O_3_ film coverage layer onto the TiO_2_ rods, the sheet-like crystal feature of the a-Bi_2_O_3_ coverage layer in the TiO_2_–Bi_2_O_3_ composite rods markedly increased the light-harvesting ability of the TiO_2_. The multi-junctions and unique sheet-like surface crystal feature of the TiO_2_/a–Bi_2_O_3_ composite rods explained their superior photoactivity than that of the TiO_2_/s–Bi_2_O_3_ composite rods herein. Figure 7b shows the Nyquist impedance plots of the TiO_2_, TiO_2_/s–Bi_2_O_3_, and TiO_2_/a–Bi_2_O_3_ rod photoelectrodes under irradiation. It has been shown that a smaller semicircular radius in the high-frequency region represents a lower electron transport resistance and a higher separation efficiency of the photogenerated electrons and holes [28]. In Figure 7b, the radius of semicircular arc of the pristine TiO_2_ rod photoelectrode is obviously larger than that of all the TiO_2_–Bi_2_O_3_ composite rod photoelectrodes, revealing the composite structure can indeed accelerate the photoinduced electron-hole pair’s separation efficiency. Moreover, the arc radius in the Nyquist curve of the TiO_2_/a–Bi_2_O_3_ photoelectrode is the smallest, implying this composite rod system had the lowest internal charge transfer resistance and can accelerate electron transfer and restrain e^-^/h^+^ recombination under light irradiation. A small arc radius and low internal charge transfer resistance for the heterogeneous structure facilitate the interfacial transfer of charges as well as the separation of charge carriers; this has been reported in TiO_2_/β–Bi_2_O_3_ nanotube array composite films via electrodeposition [29]. Figure 7c exhibits the possible equivalent circuits for a quantitative analysis of interfacial charge transfer ability of various rod samples. A similar equivalent circuit for the heterogeneous system herein has been demonstrated in previous reported BiVO_4_/BiOI and BiOI/BiOIO_3_ heterogeneous systems [28,30]. As the illustrations show, the intercept of the semicircle in the high frequency region with real axis symbolizes the solution resistance *R*_s_ and it depends on the concentration and conductivity of the electrolyte [31]. The C indicates the electric double layer capacitor and Q is the constant-phase element [32]. *R*_ct_ represents the electron transfer resistance, and it can be estimated through the fitting of arc radii of the Nyquist curves. The R_f_ represents the rod sample resistance [31]. In general, a small radius of the Nyquist curve indicates a small *R*_ct_ value for the rod samples. In the current work, the separately evaluated *R*_ct_ values of the TiO_2_, TiO_2_/s–Bi_2_O_3_, and TiO_2_/a–Bi_2_O_3_ rods are approximately 7959, 97.97 and 77.46 Ohm. The results from the PEC and EIS experiments demonstrated that the separation and migration processes of photoinduced electron-hole pairs are greatly forwarded in the TiO_2_/a–Bi_2_O_3_ composite rod system herein.

**Figure 8:**
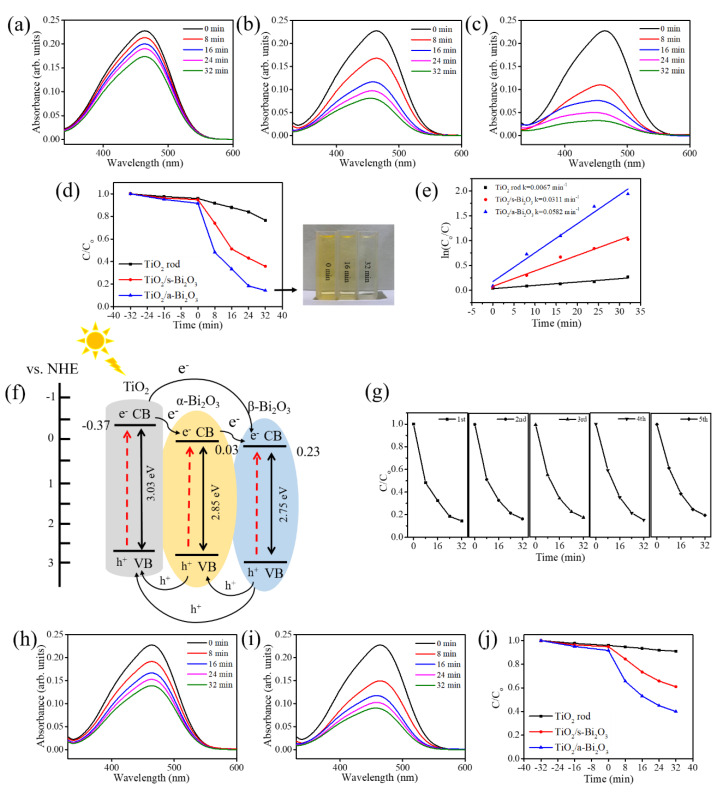
The irradiation duration dependent absorption spectra of the methylene orange (MO) solution containing various rod samples: (**a**) TiO_2_ rods, (**b**) TiO_2_/s–Bi_2_O_3_ rods, (**c**) TiO_2_/a–Bi_2_O_3_ rods. (**d**) *C*/*C*_o_ versus irradiation duration plot. The insets show the discoloration of the MO solution containing the TiO_2_/a–Bi_2_O_3_ rods under various irradiation durations. (**e**) ln (*C*_o_/*C*) versus irradiation duration plot. (**f**) The possible band alignments of the TiO_2_/a–Bi_2_O_3_ composite structure. (**g**) Recycling photodegradation tests of the MO solution containing TiO_2_/a–Bi_2_O_3_ composite rods under irradiation. The visible light irradiation time-dependent absorbance spectra intensity variation of the MO solution containing (**h**) TiO_2_/s–Bi_2_O_3_ and (**i**)TiO_2_/a–Bi_2_O_3_ photocatalysts. (**j**) *C*/*C*_o_ vs. visible light irradiation duration plots of the MO solution containing various composite rods.

The photoactivities of various rod-like photocatalysts were performed through photocatalytic decomposition experiments involving MO dyes. The pristine TiO_2_ rods were used in the comparative experiment as a photocatalytic reference to understand the improved photocatalytic activity of the TiO_2_–Bi_2_O_3_ heterogeneous rods. As depicted in Figure 8a–c, the main absorption peaks of the MO solution decreased gradually in the presence of the various rod-like photocatalysts under solar light irradiation with different durations. Comparatively, the drop in absorbance spectrum intensity was more substantial for the MO solution containing TiO_2_–Bi_2_O_3_ composite rods than that for the MO solution containing the pristine TiO_2_ rods at the given irradiation duration. The photodegradation performance of the MO solution containing various rod samples was evaluated from the concentration ratio of *C*/*C*_o_, in which *C* is the concentration of the MO solution containing the test samples after a given irradiation time, and *C*_o_ is the initial concentration of the MO solution without irradiation. The *C*/*C*_o_ vs. irradiation duration results for various rod-like photocatalysts are summarized in Figure 8d. Before irradiation, the rod-like photocatalysts were immersed in the MO solution for 32 min to reach adsorption–desorption equilibrium, and the decreased concentration of the MO solution reflected the dye absorptivity onto the surfaces of the rod-like photocatalysts. The *C*/*C*_o_ value of the MO solution decreased approximately 6% for the TiO_2_ and TiO_2_/s–Bi_2_O_3_ photocatalysts and that value was markedly dropped by approximately 9% for the TiO_2_/a–Bi_2_O_3_ photocatalyst at the given dark balance condition. This revealed that the TiO_2_/a–Bi_2_O_3_ photocatalyst exhibited more intense dye absorptivity than other rod-like photocatalysts herein. The *C*/*C*_o_ values of the MO solution containing the TiO_2_, TiO_2_/s–Bi_2_O_3_, and TiO_2_/a–Bi_2_O_3_ rods after 32 min irradiation were approximately 0.76, 0.35 and 0.14, respectively; almost 86% MO dyes are photodegraded in the solution containing the TiO_2_/a–Bi_2_O_3_ photocatalyst. Moreover, the discoloration of the MO solution containing the TiO_2_/a–Bi_2_O_3_ photocatalyst with different irradiation durations is visibly observed in the insets of Figure 8d. The MO solution containing TiO_2_/a–Bi_2_O_3_ photocatalyst became almost translucent after 32 min light irradiation; this is in agreement with the *C*/*C*_o_ result. Notably, the construction of TiO_2_–Bi_2_O_3_ heterostructures markedly enhanced the photodegradation efficiency of the TiO_2_ rods. The kinetic analysis of the MO photodegradation processes containing various rod-like photocatalysts was performed to compare the photodegradation efficiencies of various rod-like photocatalysts. The kinetic linear simulation curves of the photocatalytic MO degradation with different rod-like photocatalysts demonstrated that the degradation reactions follow an apparent first-order kinetic model at low initial concentrations. The kinetic model follows the formula ln (*C*_o_/*C*) = kt herein, where k is the first-order rate constant (min^−1^) and t is irradiation duration [8]. The k values determined for various rod-like photocatalysts are demonstrated in Figure 8e. The Bi_2_O_3_ thin coverage layer shows a significant influence on the photocatalytic degradation performance of the TiO_2_ rods towards MO dyes. In comparison with the TiO_2_ rods, the decoration of the β-phase Bi_2_O_3_ coverage layer enhanced the k value to 0.0311 min^−1^; moreover, the decoration of the α/β dual-phase Bi_2_O_3_ coverage layer substantially improved the k value to 0.0582 min^−1^, revealing more efficient enhancement in photoactivity of the TiO_2_ rod-based photocatalyst using the dual-phase Bi_2_O_3_ film. It has been shown that α/β dual-phase Bi_2_O_3_ nanofibers demonstrate a higher photoactivity to photodegrade RhB dyes than that of the single-phase constituents [33]. Essentially superior photoactivity in the α/β dual-phase Bi_2_O_3_ than that of the β-phase Bi_2_O_3_ might explained the superior photocatalytic performance of the TiO_2_/a–Bi_2_O_3_ photocatalyst herein. The possible band alignments between TiO_2_ rod and a-Bi_2_O_3_ film is shown in Figure 8f. The conduction band (CB) and valence band (VB) positions of the TiO_2_ are at −0.37 eV and 2.66 eV (vs. Normal Hydrogen Electrode, NHE), respectively [34]. The CB and VB positions of the α-Bi_2_O_3_ are at 0.03 eV and 2.88 eV (vs. NHE), respectively. Moreover, the CB and VB of β-Bi_2_O_3_ are at 0.23 eV and 2.98 eV (vs. NHE), respectively [35]. Furthermore, the type II heterojunctions formed from α-Bi_2_O_3_/β-Bi_2_O_3_, TiO_2_/α–Bi_2_O_3_, and TiO_2_/β–Bi_2_O_3_ in the TiO_2_/a–Bi_2_O_3_ photocatalyst demonstrates a synergetic effect in the substantially improved photoactivity. The suitable band alignments at the three types of heterogeneous interfaces in the TiO_2_/a–Bi_2_O_3_ photocatalyst improved the photoinduced charge separation efficiency in the composite rods. When the TiO_2_/a–Bi_2_O_3_ photocatalyst was excited by light with photon energy higher or equal to the band gaps of the Bi_2_O_3_ and TiO_2_, photoinduced electrons in the conduction band of TiO_2_ might flow to that of α-Bi_2_O_3_, then reach that of β-Bi_2_O_3_. A stepwise transfer of photoinduced electrons in the TiO_2_/a–Bi_2_O_3_ photocatalyst with a stepped heterogeneous energy band structure reduced the recombination number of photoinduced electrons. Simultaneously, photogenerated holes in the valence band of the β-Bi_2_O_3_ transfer to that of α-Bi_2_O_3_, then to that of TiO_2_. In the TiO_2_–Bi_2_O_3_ heterogeneous system, the TiO_2_ acts as a pathway for the transportation of holes. The effective separation of photogenerated carriers in the composite rods herein leads to the enhancement of their photoactivity performance. A similar design of multijunctions with a stepped band alignment configuration formed in the composite structures with three constituent components to improve their photoactivity have been reported in TiO_2_/CdS–NiO*_x_* nanorod and NiO–CdO–ZnO systems [27,36]. The possible reactions involved in the photodegradation process of the MO solution containing the TiO_2_/a–Bi_2_O_3_ photocatalyst are described below [1,2,37]:

(1) $$\alpha -Bi_{2}O_{3}+hv\;\to \;\alpha -Bi_{2}O_{3}\;\left(e^{-}\right)+\alpha -Bi_{2}O_{3}\;\left(h^{+}\right)$$

(2) $$\;\beta -Bi_{2}O_{3}+hv\;\to \;\beta -Bi_{2}O_{3}\;\left(e^{-}\right)+\beta -Bi_{2}O_{3}\;\left(h^{+}\right)\;$$

(3) $$\;TiO_{2}+hv\;\to \;TiO_{2}\;\left(e^{-}\right)+TiO_{2}\;\left(h^{+}\right)\;$$

(4) $$\;OH^{-}+h^{+}\;\to \;\cdot OH\;$$

(5) $$\;h^{+}+H_{2}O\;\to \;\cdot OH+H^{+}\;$$

(6) $$\;O_{2}+e^{-}\;\to \;\cdot O_{2}^{-}\;$$

(7) $$\;\cdot O_{2}^{-}+H^{+}\;\to \;HO_{2}\cdot \;$$

(8) $$\;2HO_{2}\cdot \;\to O_{2}+H_{2}O_{2}\;$$

(9) $$\;H_{2}O_{2}+\cdot O_{2}^{-}\;\to \;\cdot OH+OH^{-}+O_{2}\;$$

(10)  ·OH+MO → degradation products 

The hydroxyl radical ⋅OH finally formed from the above possible series reactions can decompose MO dyes directly during the photodegradation process. The photoactivity stability of the TiO_2_/a–Bi_2_O_3_ photocatalyst in photodegrading the MO solution under light irradiation was evaluated using the recycling tests as shown in Figure 8g. After five repeat test cycles, the TiO_2_/a–Bi_2_O_3_ photocatalyst retained consistent photoactivity without apparent deactivation. The retained photoactivity after cycling tests considerably promotes the practical application of this composite structure in eliminating MO dye pollutants. In order to understand the visible light-driven photodegradation effects on the formed heterogeneous systems, control groups including the TiO_2_/s–Bi_2_O_3_ and TiO_2_/a–Bi_2_O_3_ photocatalysts photodegraded towards MO solution at the same irradiation duration but with visible light irradiation were conducted for a comparison. Figure 8h,i show the time-dependent absorbance spectra intensity variation of aqueous MO solution containing TiO_2_/s–Bi_2_O_3_ and TiO_2_/a–Bi_2_O_3_ photocatalysts under visible light irradiation, respectively. It is visibly observed that the intensity of absorbance spectra deceased with visible light irradiation duration. Comparatively, the drop degree of the absorbance spectra intensity is lower than that of the MO solution containing the same photocatalysts under solar light irradiation at the same given irradiation duration (Figure 8b,c). It is supposed that the contribution of photoexcited charges from TiO_2_ because of its wide bandgap in the UV light region is prohibited to participate in MO dye photodegradation processes herein. The *C*/*C*_o_ vs. irradiation duration plots for the MO solution with two different composite photocatalysts are displayed in Figure 8j. The photodegradation degree decreased to approximately 39% and 60% for the MO solution with TiO_2_/s–Bi_2_O_3_ and TiO_2_/a–Bi_2_O_3_ photocatalysts, respectively after 32 min visible light irradiation. Although the MO solution photodegradation from TiO_2_ was restrained (referred to the result from the *C*/*C*_o_ variation with irradiation duration in Figure 8j), the contribution of the Bi_2_O_3_ coverage layer under visible light irradiation is clearly visible. Furthermore, the TiO_2_/a–Bi_2_O_3_ photocatalyst exhibited higher visible light photodegradation capability towards MO dyes than that of the TiO_2_/s–Bi_2_O_3_. The effect of the aforementioned α/β heterojunction in the a-Bi_2_O_3_ coverage layer film on photoactive performance is also clearly demonstrated in the visible light-driven MO photodegradation testes.

## 4. Conclusions

In conclusion, the rutile TiO_2_ rod templates coated with various Bi_2_O_3_ phase layers were prepared by in situ sputtering crystal growth and post-annealing procedures in ambient air. The microstructural analysis results demonstrate crystalline TiO_2_–β-Bi_2_O_3_ and TiO_2_–α/β-Bi_2_O_3_ composite rods were formed in this study. In comparison to the flat layered morphology of the β-Bi_2_O_3_ coverage film, the α/β-Bi_2_O_3_ coverage layer exhibited a sheet-like feature on the TiO_2_ rod templates. The photoresponse, EIS, and organic dye photodegradation performance results demonstrate that the TiO_2_ rod templates coated with the α/β-Bi_2_O_3_ thin layer substantially improved the photoactivity of the TiO_2_ rod templates than the TiO_2_ rod templates coated with β-Bi_2_O_3_ thin layer. The unique sheet-like surface crystal feature of the TiO_2_–α/β-Bi_2_O_3_ composite rods increased their light-harvesting ability; moreover, the formation of multi-junctions in the TiO_2_–α/β-Bi_2_O_3_ composite structure efficiently promotes separation of the photoexcited e^−^/h^+^ pairs and charge transfer ability as well as restrains recombination of the charge carriers. The results herein show that the construction of the TiO_2_–α/β-Bi_2_O_3_ composite rods via the combinational methods consisted of the hydrothermal rod growth, and sputtering and post-annealing assisted thin-film growth is promising for photoactivated devices applications.
